# The role of vitamin D in skeletal and cardiac muscle function

**DOI:** 10.3389/fphys.2014.00145

**Published:** 2014-04-16

**Authors:** Patsie Polly, Timothy C. Tan

**Affiliations:** ^1^Inflammation and Infection Research Centre, School of Medical Sciences, Faculty of Medicine, UNSW AustraliaKensington, NSW, Australia; ^2^Department of Pathology, School of Medical Sciences, Faculty of Medicine, UNSW AustraliaKensington, NSW, Australia; ^3^Cardiac Ultrasound Laboratory, Department of Cardiology, Massachusetts General HospitalBoston, MA, USA

**Keywords:** cytokines, cancer cachexia, skeletal muscle, cardiac muscle and transcriptome

## Abstract

Myopathy is a feature of many inflammatory syndromes. Chronic inflammation has been linked to pathophysiological mechanisms which implicate 1,25 dihydroxyvitamin D_3_ (1,25-(OH)_2_D_3_)-mediated signaling pathways with emerging evidence supporting a role for the vitamin D receptor (VDR) in contractile and metabolic function of both skeletal and cardiac muscle. Altered VDR expression in skeletal and cardiac muscle has been reported to result in significant effects on metabolism, calcium signaling and fibrosis in these tissues. Elevated levels of serum inflammatory cytokines, such as IL-6, TNF-α and IFNγ, have been shown to impact myogenic and nuclear receptor signaling pathways in cancer-induced cachexia. The dysregulation of nuclear receptors, such as VDR and RXRα in muscle cells, has also been postulated to result in myopathy via their effects on muscle structural integrity and metabolism. Future research directions include generating transcriptome-wide information incorporating VDR and its gene targets and using systems biology approaches to identify altered molecular networks in human tissues such as muscle. These approaches will aid in the development of novel therapeutic targeting strategies for inflammation-induced myopathies.

## Vitamin D and muscle function

Vitamin D is necessary for the maintenance of structural integrity and function of the musculoskeletal system (Pfeifer et al., 2002). Severe deficiency results in impaired bone strength and deformation i.e., rickets in children and osteomalacia in adults. Vitamin D plays a significant role in calcium homeostasis and bone metabolism through its actions on target tissues (DeLuca, 2004). Serum vitamin D levels have been correlated to muscle cell contractility, muscle strength, and postural stability (Rodman and Baker, 1978; Marcinkowska, 2001; Grimaldi et al., 2013; Girgis et al., 2014). Low serum levels have been related to proximal muscle weakness, gait disturbance, paresthesia, and discomfort within the muscles (Skaria et al., 1975; Schott and Wills, 1976; Glerup and Eriksen, 1999; Glerup et al., 2000; Pfeifer et al., 2002; Ahmed et al., 2009).

The clinical features of myopathy associated with severe vitamin D deficiency are supported by findings of abnormal histological and electrophysiological changes in muscle. Histological analysis of skeletal muscle biopsied from adults with vitamin D deficiency reveal enlarged inter-fibrillar spaces, infiltration of fat, presence of glycogen granules, fibrosis, and type II muscle fiber (fast-twitch) atrophy (Sorensen et al., 1979; Yoshikawa et al., 1979; Boland, 1986; Sato et al., 2005). Recent interest has been in reversal of some of these pathological effects of this clinical syndrome. Meta-analysis of randomized controlled trials in the elderly with low serum levels of vitamin D, demonstrated a decrease in the risk of falls following supplementation with vitamin D (Rejnmark, 2011). This outcome has been attributed to the ability of vitamin D to impact muscle fiber composition hence skeletal muscle structure. Studies in vitamin D deficient patients revealed an increase in percentage of type II fibers, a significant increase in mean type II muscle fiber diameter and area particularly of type IIa muscle fibers following treatment with 1-α-hydroxyvitaminD_3_ and calcium. However, it is still unclear if vitamin D supplementation induced formation of new type II fibers or increased transition of existing type I fibers from to type II (Sorensen et al., 1979; Sato et al., 2005). Vitamin D has also been demonstrated to increase cell proliferation and inhibit apoptosis in injured rat soleus skeletal muscle, with positive functional outcomes such as faster recovery of contraction forces (Stratos et al., 2013). The therapeutic potential of vitamin D supplementation has also recently been tested on dysferlin gene regulation and dysferlinopathies (autosomal recessive neuromuscular disorder characterized by progressive muscle wasting due to dysferlin gene mutations and a deficiency of functional dysferlin protein). Vitamin D increased dysferlin gene expression in both HL60 monocytes and skeletal muscle cells via the activation of vitamin D receptor (VDR) which binds to the dysferlin promoter; and non-genomic MEK/ERK signaling and classical genomic effects. 1,25(OH)_2_D_3_ has also been reported to suppress myotube formation by decreasing Myf5 and myogenin gene expression resulting in increased myotube diameters but reduced myostatin expression potentially alleviating the myopathic effects of muscle weakness and reduced contractile function (Luna et al., 2012).

Experiments in C2C12 cells highlight some key molecular regulatory effects of 1,25(OH)_2_D_3_ including: (1) increased expression and nuclear translocation of the VDR, (2) decreased cell proliferation, (3) decreased IGF-I expression, and (4) increased IGF-II and follistatin expression and decreasing the expression of myostatin which appeared to promote myogenic differentiation and (5) altered differentiation and myotube size. Hence, vitamin D may also be considered for use in intervention studies for muscle conditions that involve these mechanisms (Garcia et al., 2011; Girgis et al., 2014).

## The role of vitamin D receptor in muscle function

The effects of vitamin D are modulated by its receptor, therefore the expression and distribution of VDR is of significant importance. Early studies demonstrated the presence of the VDR in cultured human myoblasts and myotubes which showed a response to physiological concentrations of 1,25-(OH)_2_D_3_. VDR is also present in human skeletal muscle cells within the nuclei and has been shown to play a role in skeletal muscle development, my fiber size and morphology (Simpson et al., 1985; Costa et al., 1986; Bischoff et al., 2001; Bischoff-Ferrari et al., 2006). Skeletal muscle development requires a co-ordinated series of transcription factor and growth factor events that enable progenitor cells to undergo myoblast determination (requiring Pax3, Pax7, MyoD, and Myf5) then myoblast to myotube determination (requiring p21^Cip1^, myogenin, MEF2C and Rb) then further myotube maturation requiring innervation, MRF4, MLP) (Ludolph and Konieczny, 1995; Perry and Rudnicki, 2000; Ryhänen et al., 2003; Miyazawa et al., 2005; Washington et al., 2011). VDR and myosin heavy chain isoform was shown to co-localize in skeletal muscle biopsies in older female subjects (Ceglia et al., 2010). VDR has also been shown to impact the expression of myogenic transcriptional regulators, in particular Myf5, myogenin, E2A, and early myosin heavy chain isoforms (Endo et al., 2003; Girgis et al., 2014). C2C12 myoblasts treated with 1,25-(OH)_2_D_3_ showed increased VDR and CYP24A1 expression above endogenous levels which resulted in inhibition in cell proliferation (Srikuea et al., 2012; Girgis et al., 2014). Furthermore, inhibition of myogenic differentiation of C2C12 and G8 cell lines was also achieved with suppression of VDR expression, suggesting that myoblasts require signals transmitted through VDR for differentiation into myocytes. Myogenic differentiation likely involves the orchestration of myogenic transcription factors in skeletal muscle (Girgis et al., 2013). Vitamin D signaling may modulate p21^CIP1^ and Rb as well as myogenin, which are important in myogenic differentiation of myoblasts to myotubes (Ludolph and Konieczny, 1995; Perry and Rudnicki, 2000). Autocrine vitamin D signaling has also been reported to regulate functional effects such as contraction and remodeling in smooth muscle cells although the autocrine effects in skeletal and cardiac muscles still require characterization (Weisman et al., 2005; Maghni et al., 2007; Eggersdorfer and Stöcklin, 2013).

Effects in VDR-null mutant mice further highlight the importance of the VDR in muscle biology. Apart from the observed growth retardation, osteomalacia and systemic metabolic changes such as secondary hyperparathyroidism and hypocalcemia, these mutant mice also had abnormal muscle structure and function (Burne et al., 2005). VDR-null mutant mice displayed a progressive decrease in their muscle fiber diameters compared to those of wild-type mice, which was evident early in the postnatal period (prior to weaning) and associated with an abnormally high expression of myogenic differentiation factors. These observations suggest alterations in muscle cell differentiation pathways and thus abnormal muscle fiber development and maturation (Endo et al., 2003). Interestingly, the muscle fiber abnormalities described were diffuse without any preference for type I or II fibers, which was different to myopathy due to vitamin D deficiency where there was a predominance of type II fiber loss. Additionally, the mutant mice had a total 33% body weight reduction compared to controls at maturity; implying a post-natal role for VDR in maintaining weight (Song et al., 2003). Increased VDR expression is also correlated with regeneration (Srikuea et al., 2012), but levels of VDR appear to decrease with increasing age, which has been proposed as a potential mechanism contributing to reduced muscle strength in the Bischoff-Ferrari et al. (2004).

In the context of muscle biology, VDR mediates both non-genomic and genomic effects of vitamin D (Buitrago et al., 2001; Capiati et al., 2002). VDR knock-down experiments demonstrated that 1,25-(OH)_2_D_3_-induced p38 MAPK activity occurs through Src phosphorylation, while also reducing ERK1/2 and Akt activity. These non-genomic effects include the stimulation of transmembrane second messenger systems involving adenylyl cyclase/cAMP/PKA and PLC/DAG+IP_3_/PKC to affect contractile function and myogenesis. Furthermore, 1,25−(OH)_2_D_3_has also been reported to mediate Ca^2+^ release through voltage and store dependent calcium channels (SOC, CEE) in avian muscle cells (Santillan et al., 2004). Caveolae have also been shown to be involved in 1,25−(OH)_2_D_3_activation of in c-Src-MAPKs in C2C12 cells. Ca^2+^ influx in caveolae is triggered by the interaction between VDR with TRCP3, an integral protein of capacitative Ca^2+^ entry (CCE) (Buitrago and Boland, 2010; Buitrago et al., 2011).

###  

#### New perspectives on vitamin D, chronic inflammation, and muscle physiology

**Figure 1:**
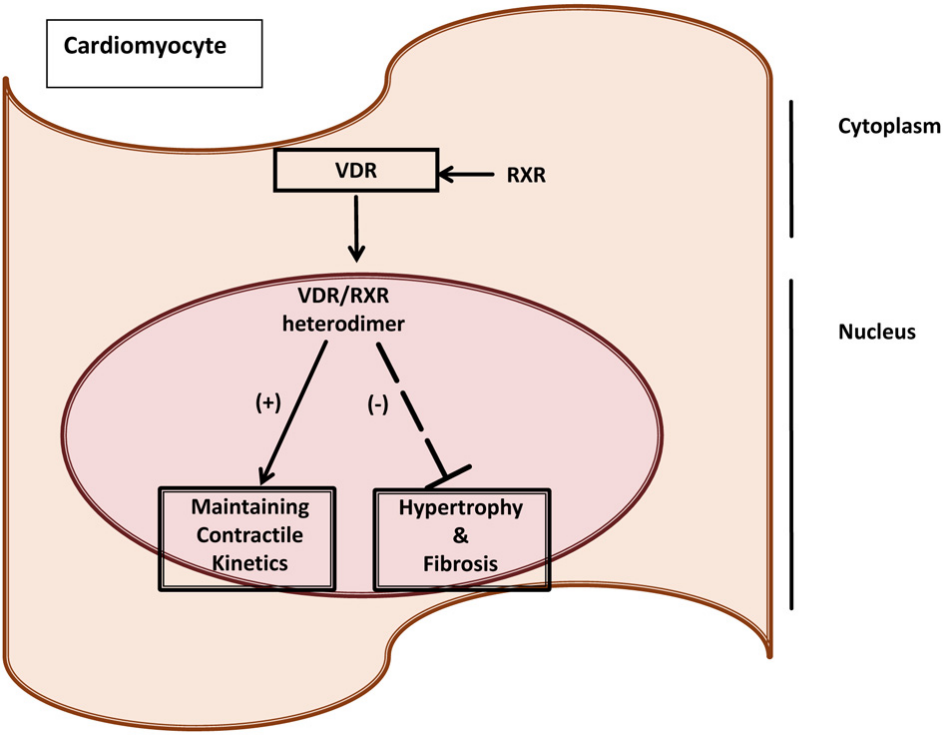
**VDR as a marker of cardiac dysfunction induced by cancer cachexia**. Vitamin D Receptor (VDR) as a molecular marker of maladaptive responses due to cancer cachexia in the heart. VDR has been linked to having cardioprotective mechanisms by maintaining contractile kinetics, and regulating fibrosis and hypertrophy (Yuan et al., 2007; Tishkoff et al., 2008; Koleganova et al., 2009).

Valuable insights into the role of vitamin D and muscle function have arisen from the study of certain pathological settings such as chronic inflammatory conditions. These conditions share phenotypic characteristics to vitamin D deficiency states and VDR-null mice. Myopathy is a feature of a number of chronic inflammatory syndromes. Chronic inflammation has been linked to pathophysiological mechanisms which implicate non-genomic and genomic 1,25-(OH)_2_D_3_-mediated signaling pathways. Skeletal muscle may be considered as having a level of plasticity, allowing it to respond to environmental, physiological and pathophysiological stimuli that elicit alterations in size, fiber-type and metabolism. Molecular factors such as insulin-like growth factors, calcineurin, desmin, Myf5, Mrf4, MyoD and myogenin have been identified as positive regulators of muscle size, while tumor necrosis factor (TNF)-α, myostatin and components of the ubiquitin pathway, have been recognized as regulators of muscle wasting. Emerging evidence supports a role for VDR in the contractile and metabolic function of both skeletal and cardiac muscle in health and disease (Figure 1). The expression of VDR and its interaction at the molecular level with proteins that are involved in inflammation, signaling and ultimately contractile function of both skeletal and cardiac muscle is of importance. *In vitro* cell culture models, *in vivo* rodent models as well as clinical studies in humans are starting to clarify the mechanisms of vitamin D action mediated via the VDR in muscle in order to enhance our understanding of their role in inflammatory mediated myopathy and muscle weakness (Girgis et al., 2012, 2013, 2014).

Exercise-induced muscle damage has been shown to increase the expression of VDR while altering gene expression of inflammatory cytokines such as interleukin (IL)-6 and TNF-α and alterations in signaling molecules involved with vitamin D signaling pathways such as phosphorylation of AMPK, p38, ERK1/2, IKK, and Iκ B simultaneously (Choi et al., 2013). An inverse relationship is generally reported for vitamin D, cancer and muscle structure and function. Alterations in metabolic status and physical activity play a role, however paraneoplastic syndromes such as cancer cachexia integrate many metabolic and catabolic molecular mechanisms which result in pathophysiological skeletal and more recently cardiac muscle effects (Choi et al., 2013). Low serum vitamin D levels are highly prevalent in advanced cancer patients with cachexia or fatigue (Dev et al., 2011). Elevated levels of inflammatory circulating factors, include C-reactive protein (CRP), a currently utilized clinical marker. The VDR axis is reported to play a fundamental role with possible association between CRP and VDR gene polymorphisms, in cancer patients with cachexia. This suggests the notion of cachexia-prone genotypes or to cachexia-resistant genotypes (Punzi et al., 2012). It has been suggested that tumor associated effects such as these may in part be addressed by nutraceutical vitamin D supplemented diets to improve vitamin D status (Endo et al., 1998; Morley, 2009; Morley et al., 2009; Strohle et al., 2010).

Data arising from the study of muscle structure and function in cancer cachexia has revealed new insights into vitamin D. Cancer cachexia is a debilitating clinical syndrome which causes up to 30% of cancer related deaths by either immobility, respiratory and/or cardiac failure (Fearon, 2008) and is characterized by weight loss; up-regulation of inflammatory markers such as IL-6, IL-1, TNF-α and interferon gamma (IFN)γ; hypercalcemia; and insulin resistance (Argiles et al., 2003; Sato et al., 2003; Jackman and Kandarian, 2004; Evans et al., 2008; Tisdale, 2009; Asp et al., 2010). The interaction between host factors and tumor cells is proposed to cause an excess production of cytokines and improper stimulation of downstream signaling molecules which results in weakness and decreased physical activity; thus highlighting the detrimental effects of cachexia on quality of life (Dahele et al., 2007). Of these cytokines, IL-6 is thought to be a key mediator of skeletal and cardiac muscle wasting in the pathogenesis of CC (Argiles et al., 2003; Haddad et al., 2005; Baltgalvis et al., 2008; Tisdale, 2009; Carson and Baltgalvis, 2010). Current treatment strategies are limited and do little to improve survival (Michael and Tannock, 1998; Mantovani et al., 2008).

More recently, we have identified a link between IL-6, the myogenic transcriptional regulator MEF2C and muscle breakdown due to CC (Shum et al., 2012). Different underlying molecular effects may also underlie the pathological changes in skeletal vs. cardiac muscle due to cancer (Shum et al., 2012; Tan et al., 2013; Shum et al., 2013; Falconer et al., in press) “Exercise genes” have now been identified in humans, which now opens the gateway for analyses that focus on the genetic basis of performance. These include the genes encoding for: the angiotensin converting enzyme, alpha-actinin 3, bradykinin, ciliary neurotrophic factor, interleukin-15, insulin-like growth factor II, myostatin and the VDR which have been proposed to play a role in inter-subject variability in muscle strength or size. Current data is only available from healthy subjects, hence genetic variability that may account for these effects still requires further analysis particularly in the context of muscle disease (Stewart and Rittweger, 2006). Furthermore, conversion toward a fatigue prone, type II skeletal myofiber phenotype has been observed due to cancer, which potentially makes this condition treatable with vitamin D.

***Cardiac muscle effects due to cancer cachexia.*** Cardiac weight loss is a relatively unreported feature in cancer cachexia although autopsy studies revealed that “cardiac atrophy” is a prominent feature in advanced cancer patients (Hellerstein and Santiago-Stevenson, 1950). Recent studies have demonstrated that the reversal of cardiac and skeletal muscle weight loss increased longevity in mouse models of cancer cachexia, implying that these effects on the heart may contribute to poor prognosis in cancer patients (Zhou et al., 2010). The molecular basis of this cardiomyopathy induced by cancer cachexia is unclear. We and others have established the IL-6 driven, colon 26 (C26) carcinoma cachexia mouse model to study cancer cachexia (Tanaka et al., 1990; Asp et al., 2010; Zhou et al., 2010; Shum et al., 2012). The C26 model demonstrates significant body wasting, has no metastases to the heart, thus effects seen are largely due to the tumor or the host-tumor response (Matsumoto et al., 1999; Schwarzkopf et al., 2006; Strassmann et al., 1992). Features of cardiac wasting in the end stages of cachexia (i.e., 20–25% body weight loss) observed in C26 and other cachectic animal models include: heart weight loss; marked fibrosis; oxidative modifications; reduced expression of contractile apparatus proteins; no increase of apoptosis; and lower ejection fraction (Fukuda et al., 2009; Springer et al., 2009; Marin-Corral et al., 2010; Tian et al., 2010; Shum et al., Unplublished Data). Genes that mediate muscle atrophy such as atrogin-1 and Murf-1, were unaltered in the heart unlike skeletal muscle; implying that cardiac wasting occurs via different molecular pathways (Zhou et al., 2010; Shum et al., 2013; Unplublished Data). Vitamin D and its gene effects in the context of functional consequences have been described in skeletal muscle cell culture models, cardiac muscle and smooth muscle (Meems et al., 2011; Girgis et al., 2014). However, the roles of VDR and 1,25-(OH)_2_D_3_ need further characterization in the context of muscle wasting due to cancer cachexia (Figure 1).

***Vitamin D and cardiac pathology.*** Vitamin D and its analogs may potentially have palliative effects in the cardiovascular system. Long term exposure to angiotensin II has been shown to induce hypertension, cardiac hypertrophy, activation of the hypertrophic fetal gene program atrial natriuretic peptide (ANP), B-type natriuretic peptide and alpha skeletal actin gene expression), increased expression of the pro-hypertrophic modulatory calcineurin inhibitor protein 1 (MCIP 1), and increased fibrosis with augmented procollagen 1 and 3 gene expression. Co-administration of paricalcitol (a vitamin D analog with agonist properties) in an animal model of non-renin-dependent cardiac hypertrophy partially reversed the reported AII-dependent effects. Interestingly, the effects of agonist-bound vitamin D receptor appeared to elicit potent anti-hypertrophic activity in this model of cardiac hypertrophy. The anti-hypertrophic activity appears to be at least partially intrinsic to the cardiac myocyte and may involve suppression of the MCIP 1 protein (Chen and Gardner, 2013). Though the cardiovascular system is not thought to represent a classical target for 1,25-(OH)_2_D_3_ and retinoic acid (RA), it is clear that both cardiomyocytes and vascular smooth muscle cells respond to these nuclear receptor hormones (NRHs) with changes in growth characteristics and gene expression (Figure 1). These NRHs suppress many of the phenotypic correlates of endothelin-induced hypertrophy in a cultured neonatal rat cardiac ventriculocyte model. Each of these NRHs reduced endothelin-stimulated ANP secretion in a dose-dependent manner and when the two were used in combination, they proved to be more effective than when either NRH was used alone. 1,25-(OH)_2_D_3_ abrogated the increase in cell size seen after endothelin treatment. These findings suggest that liganded vitamin D and retinoid receptors are capable of modulating the hypertrophic process *in vitro* and that agents acting through these or similar signaling pathways may be of value in probing the molecular mechanisms underlying hypertrophy (Wu et al., 1996) (Figure 1).

#### Transcriptome-wide effects and muscle

Recently, transcriptome-wide approaches have been applied to muscle in order to get a global view of changes that occur due to various stimuli, for example structural vs. metabolic. The transcriptional profile of VDR mRNA isoforms has been examined for differences in bone, cartilage and paravertebral muscles between tissues from curve concavity and convexity. VDR was differentially expressed in paravertebral muscles in patients with juvenile idiopathic scoliosis (JIS) and adult idiopathic scoliosis (AIS). The VDRl isoform appears to contribute to curve concavity in paravertebral muscles. Furthermore, muscular transcriptome differentiation was evident between curve concavity and convexity in JIS patients. Tob2 and MED13 gene expression in paravertebral muscles appear to differentiate the two types of idiopathic scoliosis (Nowak et al., 2012).

Gene expression has been examined in skeletal muscle tissue of obese insulin-resistant subjects before and after a euglycemic-hyperinsulinemic clamp to determine the pathogenesis of insulin resistance. Differential gene expression was demonstrated for enzymes, transcription, and translation regulators, transporters, G protein-coupled receptors, cytokines, and ligand-dependent nuclear receptors. Metabolic pathways that incorporated, inflammatory signaling and nuclear receptors were also significantly different. These included LXR/RXR activation, VDR/RXR activation, interleukin IL-8, acute phase response, IL-10, triggering receptor expressed on myeloid cells 1, peroxisome proliferator-activated receptor, G-beta/gamma and hepatocyte growth factor and IL6 signaling (Rudkowska et al., 2013).

Comparisons between transcriptomes and proteomes in muscle tissues and activated CD4+ and CD8+ T lymphocytes (T-cells) analyzed using Affymetrix microarrays and mass spectrometry, from type 2 diabetes (T2DM) subjects and matched non-diabetic controls, demonstrated reduced gene expression for insulin receptor (INSR), VDR, insulin degrading enzyme, Akt, insulin receptor substrate-1 (IRS-1), IRS-2, glucose transporter 4 (GLUT4), and enzymes of the glycolytic pathway in the T2DM subjects compared controls. Increased gene expression was shown for plasma cell glycoprotein-1, TNFα, and gluconeogenic enzymes in T2DM subjects. Observed alterations in transcriptomes and proteomes between muscle and activated T-cells of T2DM were comparable suggesting a more global molecular basis for insulin resistance (Stentz and Kitabchi, 2007).

#### Conclusion and perspectives

There is now clear evidence supporting a significant role for vitamin D in the biology and function of skeletal and cardiac muscle. Current evidence outlines a number of effects of vitamin D on these muscle types including intracellular calcium handling, differentiation and contractile protein composition. However further study using novel investigative strategies is still warranted to better delineate the role and functions of vitamin D in muscle. The molecular interplay between cytokine signaling, VDR expression, genetic variability in patients with myopathy due to chronic inflammatory conditions such as cancer cachexia may reveal the molecular basis for changes that have been observed in skeletal and cardiac muscle. Early transcriptomic studies on the effects of cytokines in muscle wasting due to cancer cachexia have provided clues regarding potential molecular mechanisms induced by cytokines that drive muscle wasting which may potentially also implicate vitamin D mediated transcriptional mechanisms although this still remains to be defined. A better characterization of the role of VDR in the context of inflammation-mediated muscle wasting and weakness may also potentially translate into significant clinical applications by informing nutraceutical approaches using vitamin D supplementation as a potential strategy for reversing muscle wasting.

### Conflict of interest statement

The authors declare that the research was conducted in the absence of any commercial or financial relationships that could be construed as a potential conflict of interest.
